# Porous Carbon Electrode Made of Biomass DNAs for High-Efficiency Quasi-Solid-State Supercapacitor

**DOI:** 10.3390/nano15040304

**Published:** 2025-02-17

**Authors:** Samanth Kokkiligadda, Surya Kiran Ampasala, Yeonju Nam, Jeonghun Kim, Suk Ho Bhang, Soong Ho Um

**Affiliations:** 1 School of Chemical Engineering, Sungkyunkwan University, Suwon 16419, Gyeonggi-do, Republic of Korea; surya99@skku.edu (S.K.A.); sukhobhang@skku.edu (S.H.B.); 2 Department of Physics, Sungkyunkwan University, Suwon 16419, Gyeonggi-do, Republic of Korea; room2093@skku.edu; 3 Progeneer Incorporation, #1002, 12, Digital-ro 31-gil, Guro-gu, Seoul 08380, Republic of Korea; jhkim@progeneer.com; 4 Biomedical Institute for Convergence at SKKU (BICS), Sungkyunkwan University, Suwon 16419, Gyeonggi-do, Republic of Korea; 5 Institute of Quantum Biophysics (IQB), Sungkyunkwan University, Suwon 16419, Gyeonggi-do, Republic of Korea; 6 SKKU Advanced Institute of Nanotechnology (SAINT), Sungkyunkwan University, Suwon 16419, Gyeonggi-do, Republic of Korea

**Keywords:** biomass DNA, carbon electrode, porosity, supercapacitor

## Abstract

Since companies have declared their commitment to operating with 100% renewable energy, developing electrical storage systems using natural eco-friendly resources is in full swing. Efforts to replace existing materials in core electrode materials are accelerating, but the use of toxic chemicals in the complex production process is decreasing its value. This study presents a unique porous carbon electrode made of pure biomass DNA wastes synthesized simply via a single step of hydrogelation-calcination without activation through carbonization. Electrochemical analysis of the electrodes revealed energy storage performance with an outstanding specific capacitance of 563.34 F g^−1^ at 1 A g^−1^. The QSSC exhibited an energy density of 13.05 Wh kg^−1^ and a power density of 486.67 W kg^−1^. It was connected to a solar panel for renewable energy storage and successfully powered a digital clock and LEDs (Light Emitting Diode), demonstrating the potential of advanced sustainable and cost-effective energy storage solutions.

## 1. Introduction

The energy sector is experiencing significant transformation as traditional nonrenewable resources like gas, coal, and oil become increasingly depleted. This shift is driven by urgent challenges such as climate change and environmental pollution. To build a sustainable future, there is a growing emphasis on adopting renewable energy storage systems generated from solar, wind, water, and biomass [1,2]. This transition is essential for our energy needs while reducing environmental impacts and ensuring a healthier planet for future generations. Moreover, the escalating demand for portable energy devices has intensified efforts in research and development. An emerging and promising direction is the exploration of biomaterials for both energy harvesting and storage purposes. Activated carbon is commonly employed in energy storage applications. Yet, its commercial accessibility is often accompanied by high costs and time-consuming synthesis procedures. A growing interest lies in a different approach integrating biologically sourced materials into this carbon structure [3,4,5]. This novel approach offers promises for enhancing energy storage efficiency while also reducing costs and streamlining production. The use of biomaterials in this context is particularly attractive due to their non-toxic nature, economic feasibility, and universal availability. Biomass has gained significant attention as a precursor for carbon materials because of its affordability, accessibility, and sustainability when compared to conventional carbon sources [6,7,8,9]. It can be easily converted into conductive carbon, providing a cost-effective and efficient method for recycling biomass. While some carbon materials, such as carbon nanotubes (CNTs) and graphene, can store a lot of energy, but their complex preparation processes and limited raw material supply make them highly expensive. This significantly restricts their potential for large-scale applications.

However, the path of energy conversion encounters a significant obstacle in the requirement for effective energy storage mechanisms. Without such dependable solutions, we cannot fully utilize the potential of renewable energy sources. This is where supercapacitors (SC) come into the picture. They become a popular choice for energy storage because they are affordable, charge quickly, are easy to make, have high power, and last a long time [10,11,12,13]. For many years, carbon-based materials have been used as negative electrodes in hybrid supercapacitors because they store charge mainly through electrical double-layer capacitance (EDLC). Their performance depends on the surface area, the larger the surface, the better the interaction between the electrode and electrolyte, leading to improved energy storage. Biomass-based activated carbon (AC) has been made from sources like waste perilla frutescens, camellia, and rice husk with capacitance values of 270, 125, and 163 F g^−1^, respectively [14,15,16]. However, the performance of activated carbon from biomass was lower than that of chemically synthesized carbon electrodes. To improve this, it is important to choose biomass sources that naturally contain polymers. For example, DNA from salmon fish waste has been used to create a negative electrode through a simple one-step hydrogel carbonization process.

In this study, a novel synthesis of biomass derived from DNA source to explore their potential use as electrodes in electrochemical supercapacitor. The abundance and low cost of marine biology salmon fish waste extracted DNA makes it an excellent candidate for producing carbon material offering an alternative to commercially available carbons. The activated carbon synthesized from DNA in single step without any activation exhibited a capacitance of 563.34 F g^−1^. In this study, a quasi-solid-state supercapacitor (QSSC) was developed using environmentally friendly source DNA. The device was constructed with DC-900 serving as the electrode and utilized a Deoxyribonucleic acid (DNA)-potassium hydroxide (KOH) as the electrolyte. The resulting QSSC demonstrated excellent electrochemical performance. Additionally, to showcase its practical applications, solar energy harvesting was integrated with the QSSC.

## 2. Materials and Methods

### 2.1. Materials

Deoxyribonucleic acid (DNA) fibers obtained from GEM corporation Shiga, Japan. 0.5 M sulfuric acid (H_2_SO_4_), KOH pellets, and NMP (N-Methyl-2-Pyrrolidone) were purchased from Sigma-Aldrich Inc., Seoul, South Korea. Activated carbon (AC) was sourced from MTI Corporation, Seoul, South Korea. All chemicals obtained were of analytical grade and were used as received without any additional purification. The experiments utilized distilled water (DI) with a resistivity of 18.2 MΩ.

### 2.2. Preparation of Porous Carbon by One Step from Biomass DNA

Salmon sperm DNA (Deoxyribonucleic acid) weighing 0.6 g was mixed with 20 mL of deionized water over a 2 h duration at 90 °C until completely dissolved by employing a magnetic stirrer. The DNA solution was immediately poured into a 50 mL conical tube and left to incubate at 4 °C temperature for 1 h. During the cooling process, the solution solidified, forming a homogeneous hydrogel. Further, these DNA hydrogels were kept in a freeze dryer for 36 h at −50 °C. Later, the collected ultra-lightweight DNA hydrogel foams were calcinated in a tube furnace at desired temperatures (600–1000 °C) in a nitrogen atmosphere. The obtained resultant black powder was further washed by using 0.5 M H_2_SO_4_, followed by DI water until pH reached to neutral level to remove excess Na, P, and impurities. The black powder was dried in an oven overnight at 50 °C; resultant powders were collected and further characterizations.

### 2.3. DNA Electrodes Preparation

DC-x electrodes (where x = 600, 800, 900, and 1000) were directly employed as working electrodes. To create the working electrode for DC, a mixture of active material (DC), super P carbon, and polyvinylidene fluoride (PVDF) binder was prepared in a weight ratio of 70:20:10 and thoroughly ground. This mixture was then combined with a few drops of NMP to form a slurry, which was coated onto a 1 × 1 cm^2^ area of cleaned carbon cloth. The coated electrode was dried at 120 °C for 6 h. AC electrodes were measured at 2.45, 2.48, 2.36, and 2.52 mg, respectively. These electrodes were then utilized as working electrodes in the investigation of their electrochemical properties. Conventional beaker-type three-electrode setups were utilized, with Pt wire serving as the counter electrode and Ag/AgCl as the reference electrode, all immersed in a 1 M aqueous KOH solution. Various analyses including cyclic voltammetry (CV), galvanostatic charge–discharge (GCD), and electrochemical impedance spectroscopy (EIS) with a frequency range from 100 kHz to 0.01 Hz were conducted for all the electrodes.

### 2.4. DNA/KOH Gel Electrolyte Preparation

To synthesize a DNA-KOH gel electrolyte, 5 wt.% of salmon DNA was dissolved in 10 mL of deionized water under stirring at 90 °C temperature until homogeneous solution. Additionally, a 1M KOH solution was prepared by dissolving the appropriate amount of potassium hydroxide in deionized water. The KOH solution was gradually added to the DNA solution while stirring continuously allowing the DNA to interact with the KOH and form a viscous until gel-like consistency. The mixture should be stirred until uniform. The solution was heated gently to 60 °C to promote complete gelation. The DNA/KOH gel solution was poured into a 90 × 90 mm Petri dish, and it was cooled down at room temperature for 2~4 h until the gel was fully solidified. After cooling, the gel was rinsed with a KOH solution to stabilize it. The resulting DNA-KOH gel electrolyte was ready for electrochemical applications.

### 2.5. Characterizations

The surface morphology of the synthesized samples was examined using a field-emission scanning electron microscope (FE-SEM, JSM7000F, JEOL, Tokyo, Japan). Elemental composition analysis was performed via energy-dispersive X-ray (EDX) spectroscopy. To evaluate the specific surface area and pore size distribution, the samples were analyzed using the Brunauer–Emmett–Teller (BET) method and the Barrett–Joyner–Halenda (BJH) method, respectively, based on nitrogen adsorption–desorption isotherms. The crystal structure was assessed by X-ray diffraction (XRD) using a Rigaku MiniFlex600 (Tokyo, Japan) equipped with Cu Kα radiation (λ = 1.5406 Å) and a Ge monochromator. The oxidation states of the elements in the optimized sample were determined by X-ray photoelectron spectroscopy (XPS) using an ESCALAB250 system with a K-alpha source (Thermo Electron). Electrochemical performance was investigated through cyclic voltammetry (CV), galvanostatic charge–discharge (GCD) measurements, and electrochemical impedance spectroscopy (EIS) in a 1 M KOH electrolyte. These tests were conducted using a BioLogic VSP 3e electrochemical workstation in a traditional three-electrode setup at room temperature.

## 3. Results and Discussion

### 3.1. Fabrication of a Carbon Electrode Made of Biomass DNA Wastes

**Figure 1:**
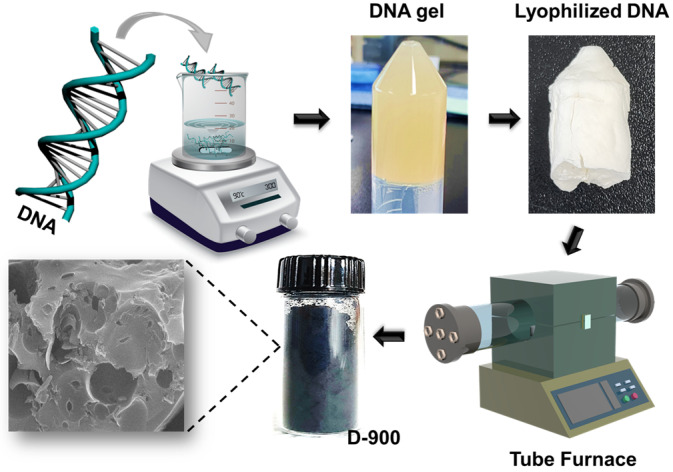
Schematic illustration of the fabrication process for DC-x porous carbon derived from pure DNA.

Salmon sperm DNA, which is discarded by tens of millions of tons each year, was used as a proof model in this study. DNA was homogeneously hydrogelized in a cooling–heating iteration process and became an ultra-light porous sponge after lyophilization and calcinated, in turn becoming an activated carbon electrode in black powder and here labeled as a carbon electrode made of DNA (DC) (Figure 1). Surface morphology, porosity, surface area, and elemental analysis were performed while operating at different calcination temperatures of 600~1000 °C (Figures S1–S5). A smooth surface with small pores appeared at 800 °C, while an increase in temperature to 900 °C yielded a micro-size porous structure (Figures S1 and S2). However, at 1000 °C, a meso- and microporous structure formed, enhancing surface accessibility (Figures S1 and S2). EDX spectroscopy on DC-900 confirms the presence of C, O, and N, indicating retained sugar-phosphate and hydroxyl groups. The X-ray analysis (Figure S4a) shows broad peaks around 2θ ≈ 22°, 24.45°, and 43.70°, indicating the amorphous structure of carbon in all DC electrode materials [17]. These peaks correspond to the (002) and (100) planes, suggesting parallel stacking of carbon layers and a honeycomb-like sp2 hybridized carbon structure [18,19].

### 3.2. Analysis of a Carbon Electrode Made of Biomass DNA Wastes

**Figure 2:**
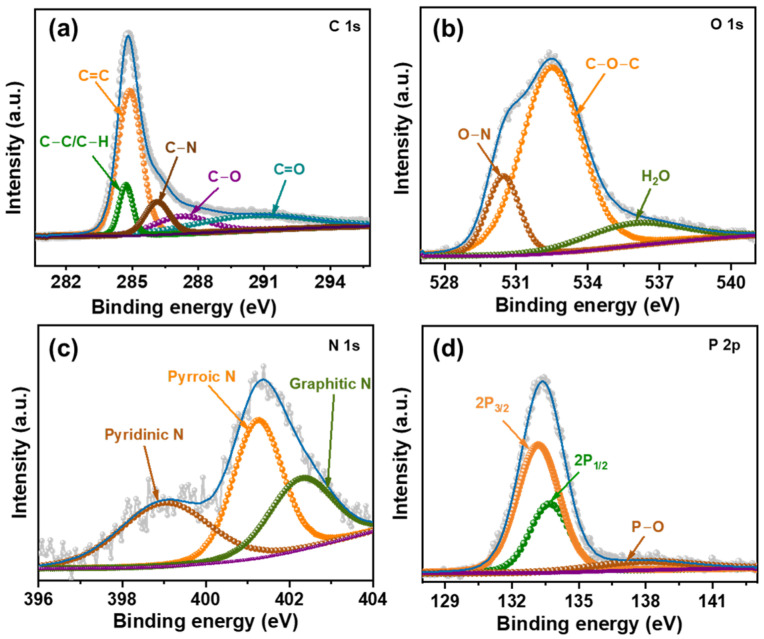
XPS analysis of DC-900 with core-level spectra of each element. (**a**) High-resolution C 1s spectrum showing deconvoluted peaks corresponding to different carbon bonding states. (**b**) O 1s spectrum indicating various oxygen functional groups. (**c**) High-resolution N 1s spectrum revealing the nitrogen bonding states. (**d**) High-resolution P 2p spectrum depicted the phosphorus chemical states and bonding environment.

To examine the chemical composition and the retention of occurring ions in DC-900 XPS analysis was conducted as shown in Figure 2 and Figure S4b. The results obtained are consistent with those from the EDX analysis. Figure 2 provides the Gaussian fitting of the core-level XPS elemental spectra. The high-resolution C 1s spectra were deconvoluted into five distinct peaks at binding energies of 284.73 eV is attributed to C–C/C–H (sp^3^ carbon), while the presence of C=C (sp^2^ carbon) is observed at 284.88 eV, suggesting the formation of graphitic structures. The C–N bond found at 286.13 eV along with the peak at 287.33 eV corresponds to C–O reflecting oxygen-containing functional groups such as hydroxyls. Finally, the peak around 289.79 eV is attributed to C=O attributed to adventitious carbon species [20,21,22]. The N1s spectra revealed peaks at 399.08, 401.28, and 402.38 eV, corresponding to pyridinic-N, pyrrolic-N, and graphitic-N functional groups, respectively [23,24]. These nitrogen-containing groups are often associated with structural stability and may contribute to the formation of functional active sites within the porous carbon structure which potentially enhance the materials electrochemical properties. The O 1s spectrum exhibited three peaks at binding energies of 530.48, 532.42 assigned to O–N, C–O–C functional groups, respectively, and the broad peak at 536.38 eV may be caused by water adsorbed on the sample surface [25,26,27]. These oxygen-containing groups can positively influence the wettability and ion transport of the material. Additionally, the high-resolution P 2p spectra were deconvoluted into three distinct peaks at binding energies of 133.11 eV and 133.89 eV, corresponding to the P 2p_3_/_2_ and P 2p_1_/_2_ components of phosphate species (PO_4_^3−^) respectively, indicating the presence of phosphorus in oxidized states. The peak at 137.5 eV is attributed to highly oxidized phosphorus species or may result from surface oxidation during carbonization [28,29,30,31]. The presence of phosphorus species may stabilize the structural framework and introduce additional active sites, which can further contribute to improved electrochemical performance.

### 3.3. Electrochemical Analysis on the Porous DNA-Based Carbon Electrodes

**Figure 3:**
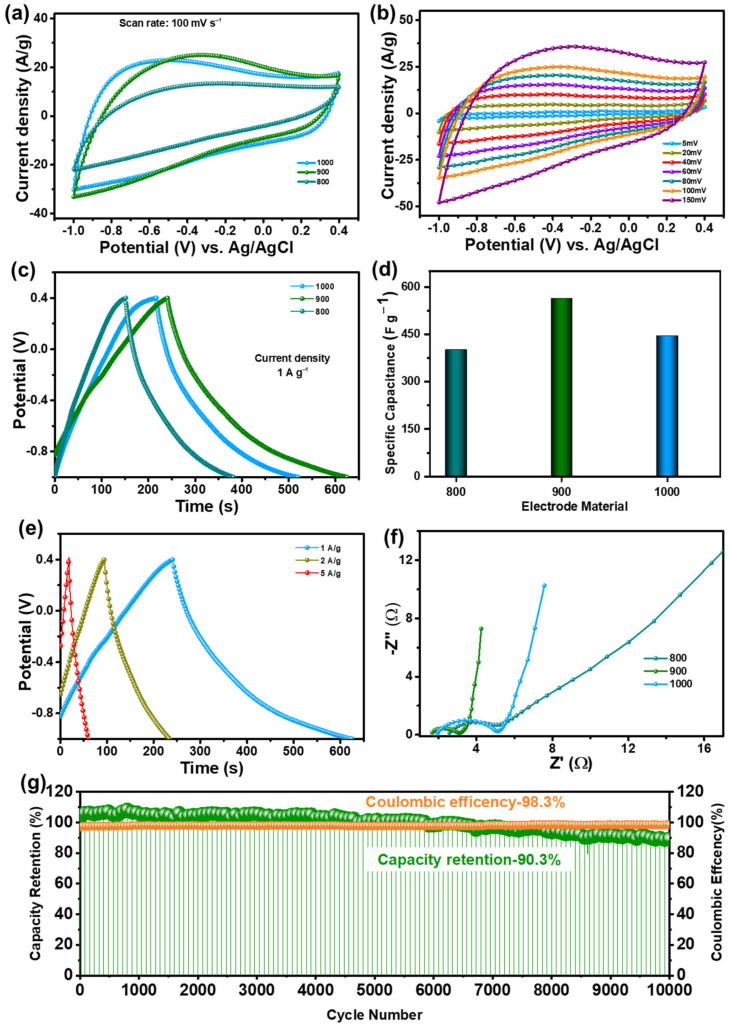
Depicted various electrochemical characterization results for the DC-x electrodes: (**a**) Comparative cyclic voltammetry (CV) curves at a constant scan rate of 100 mV s^−1^, (**b**) CV curves at different scan rates specifically for the DC-900 electrode, (**c**) the Comparative galvanostatic charge–discharge (GCD) curves at a constant current density of 1 A g^−1^, (**d**) specific capacitance values at the same current density for the DC-x electrodes, (**e**) GCD curves at different current densities specifically for the DC-900 electrode, (**f**) comparative electrochemical impedance spectroscopy (EIS) plots for the DC-x electrodes, (**g**) long-term cyclic stability of the DC-900 electrode measured at 10 A g^−1^ over 10,000 cycles.

Electrochemical analysis was conducted on DC-x electrodes with a fixed voltage range from −1 V to 0.4 V relative to the Ag/AgCl reference electrode for CV measurements at varying scan rates to analyze the current response. Galvanostatic charge–discharge (GCD) measurements were carried out at various current densities, while Electrochemical Impedance Spectroscopy (EIS) plots were also generated. A standard three-electrode electrochemical setup in a 50 mL beaker was utilized to evaluate the energy storage properties of DC-900 using a 1 M KOH electrolyte solution, with comparisons made against other prepared DC-x electrodes. Figure 3a shows a comparison of CV curves for DC-x electrodes acquired at a consistent scan rate of 100 mV s^−1^. The CV curve depicted in Figure 3b displays a distinct quasi-rectangular shape along broad peak suggesting the coexistence of electric double-layer capacitance (EDLC) and pseudo capacitance in DC-900 electrode. For comparison, the CV curves of DC-800 and DC-1000 are depicted in Figure S6a,b. This phenomenon is believed to result from the synergistic influence of nitrogen and phosphate atoms within the carbon matrix. Furthermore, DC-900 demonstrates the largest loop area among these CV curves, signifying exceptional capacitive behavior. Figure 3c shows the GCD curves of the synthesized DC-x materials at a constant current density of 1 A g^−1^ for comparison. All synthesized electrode materials show high symmetry with minimal distortion throughout the process, which is attributed to the reversible redox reactions occurring during charge and discharge. Additionally, DC-900 shows the longest discharge duration among the DC-x electrodes, which confirm its enhanced capacitive performance which aligns with the CV results. Figure 3d represents a bar chart depicting specific capacitances of each DC-x electrode measured at a fixed current density of 1 A g^−1^. The DC-900 electrode attains highest specific capacitance of 563.34 F g^−1^ while the DC-1000 and DC-800 specific capacitances with 445.63 F g^−1^ and 401.22 F g^−1^, respectively.

The GCD results exhibit strong consistency with the previously discussed CV data. Figure 3e and Figure S6b,d display GCD measurements executed at different current densities ranging from 1 to 10 A g^−1^ for all DC-x electrodes to evaluate their efficiency. The Nyquist plot provides electrochemical impedance spectroscopy (EIS) measurements. Figure 3f shows the Nyquist plots of all the DC-x electrodes for comparison. Notably, a high-frequency semicircle is present only in the DC-800 electrode, which is not observed in the DC-900 and DC-1000 electrodes. The linear response in the lower frequency region corresponds to the Warburg impedance, indicating a diffusion-limited process. For DC-900, the line shows a greater leftward inclination than the other electrodes suggesting improved conductivity, enhanced electron transfer kinetics, and more efficient ion diffusion at the electrode-electrolyte interface. Furthermore, to test the cyclic stability of the DC-900 electrode was assessed using galvanostatic charge–discharge (GCD) at 10 A g^−1^ over 10,000 cycles was shown in Figure 3g. The DC-900 electrode exhibited high-capacity retention of 92.4%, demonstrating its structural stability and ability to sustain charge storage over prolonged cycling. Additionally, a Coulombic efficiency of 98.5% was achieved indicating efficient charge transfer with minimal energy loss. These results confirm the materials excellent electrochemical reversibility and durability, making it a promising candidate for long-term energy storage applications in supercapacitors.

### 3.4. QSSC Device Equipped with DNA-Based Carbon Electrodes

**Figure 4:**
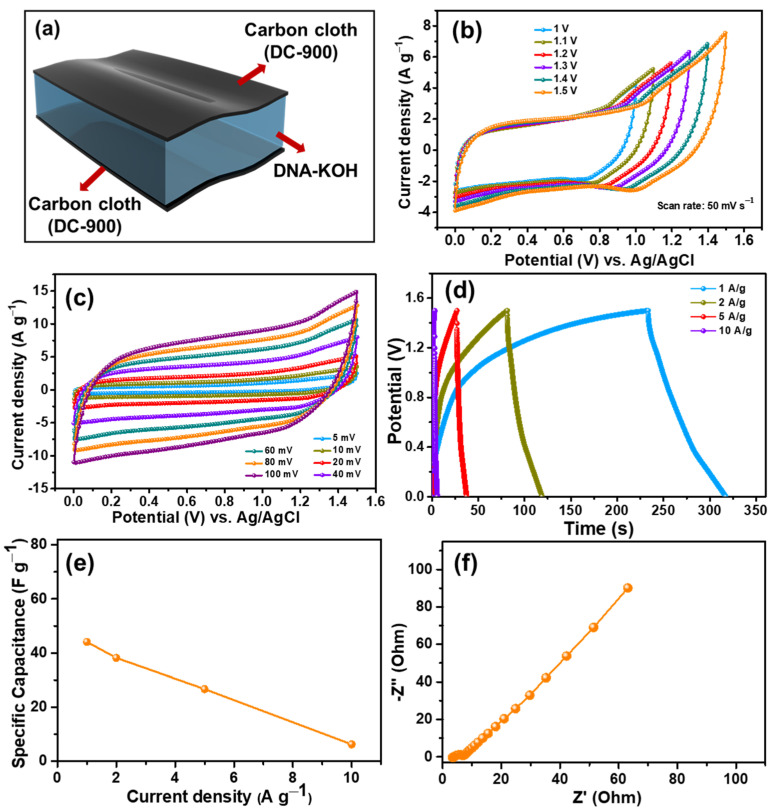
QSSC device performance: (**a**) schematic representation of QSSC device, (**b**) CV curves measured at different potential windows from 1–1.5 V under the scan rate of 50 mV s^−1^, (**c**) CV curves of the QSSC device at different scan rates 5 mV to 150 mV s^−1^, (**d**) GCD curves at different current densities, (**e**) specific capacitance values at individual current densities, (**f**) EIS plots.

To evaluate the real-time viability of the DC-900 electrode, a quasi-solid-state symmetric supercapacitor (QSSC) device equipped with DC-900 in DNA electrolyte was constructed (Figure 4a).

The CV analysis of the DC-900 electrode in a three-electrode setup exhibits nearly rectangular curves, characteristic of dominant electric double-layer capacitance (EDLC) behavior, within the potential window of −1.0 to 0.4 V. As a result, the ideal operating potential for the QSSC device was predicted to be around 1.5 V. Cyclic voltammetry (CV) measurements were performed over a potential range of 0 to 1.5 V at a fixed scan rate of 50 mV as shown in Figure 4b. The measurements obtained indicate a hybrid electrochemical energy storage mechanism in the QSSC confirming a stable potential window at 1.5 V. Figure 4c represents the CV curves of the QSSC recorded at different scan rates ranging from 5 to 100 mV s^−1^ indicating charge storage mechanisms (EDLC mechanisms). Additionally, the area of CV curves gradually increases with escalating scan rates. The GCD curves were obtained for the QSSC at different current densities ranging from 1 to 10 A g^−1^ as shown in Figure 4d. Remarkably, even under a high current density of 10 A g^−1^, the QSSC maintained its charge–discharge duration, indicating exceptional columbic efficiency. The specific capacitance of the QSSC was assessed by examining the discharge time and then plotted as a function of the corresponding current densities in Figure 4e. The device exhibits a specific capacitance of 44.06 F g^−1^ at a current density of 1 A g^−1^. Electrochemical impedance spectroscopy (EIS) measurements were performed on the QSSC device shown in Figure 4f.

**Figure 5:**
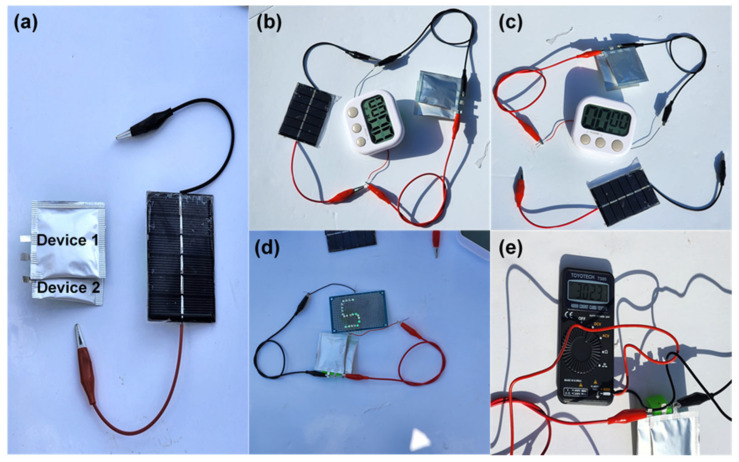
(**a**,**b**) Photograph representation of self-powered energy storage with two series connected QSSCs and solar panels intersecting to realize more eco-friendly charging and discharging and (**b**,**c**) the charging process of the QSSC device using a solar panel under natural sunlight and the operation of the digital clock. (**d**) The illumination of green LEDs. (**e**) Output voltage of the QSSC device after disconnecting from the solar panel.

Activated carbon, which is mainly used in electrical storage devices, is manufactured through a complex process using toxic chemicals. Considering environmental protection and energy economy, we are looking for a highly efficient porous activated carbon substitute for biomaterial raw materials, which is theoretically easily found in nature and suggested to be more efficient than conventional ones. There have been many suggestions, but the problems of efficiency and simplification of the process steps were not always good (Table S1). In this study, this was overcome and realized. A porous carbon structure with a large specific surface area and uniform pore distribution using biomass DNA wastes discarded every year in huge amounts was designed and fabricated to provide a supercapacitor electrode with low charge transfer resistance, high specific capacitance, and excellent charging and discharging efficiency. An integrated system with two serially connected QSSCs and a solar energy harvesting module shows potential for next-generation energy storage applications (Figure 5).

To explore the practical viability of future self-sustaining energy storage systems, a rechargeable DC-900-based QSSC was integrated with a solar energy harvesting setup, as shown in Figure 5. Solar energy, known for its universal availability and affordability, can be effectively converted into electricity through solar panels. The integration of such energy harvesting systems outdoor, mobile vehicles can generate significant electrical output, creating a demand for efficient energy storage devices to capture this energy for immediate use. The conversion of solar energy into stored energy could enable extended utilization of renewable resources, even during absence of sunlight. Supercapacitors have attracted considerable attention due to their advantages among energy storage components. This study focuses on utilizing a fabricated QSSC device for solar energy storage promoting the incorporation of renewable energy sources into diverse applications. Figure 5a presents a photographic representation of two QSSC devices connected in the series integrated with the solar panel. Upon exposure to sunlight the solar panel quickly charges the QSSC device to the desired voltage, as shown in Figure 5b. The charged QSSC devices subsequently power the digital clock display continuously as shown in Figure 5c, demonstrating a practical application of solar charging for electronic devices. Additionally, the QSSC devices can be connected to LEDs once the desired charging potential is reached as depicted in Figure 5d. Using solar-generated electricity, the QSSC device is rapidly charged to the target voltage (3 V) within 15 s (Figure 5e). After disconnecting the solar panel, the QSSC devices are then connected to the digital clock display and LEDs. This integrated design from energy harvesting to storage could pave the way for the development of self-powered charging systems for various electronic applications.

The selection of salmon DNA as a model is not arbitrary but based on its unique polymer content, particularly its rich nitrogen composition, which plays a key role in enhancing the electrochemical performance of carbon materials. Nitrogen-doped carbons have demonstrated improved capacitance and conductivity due to the presence of nitrogen heteroatoms which enhance surface interactions during electrochemical processes. While DNA might seem unconventional, its utilization aligns with the broader strategy of finding biomaterials that offer unique advantages like improved conductivity and functional group availability, which are harder to achieve with other biomass sources. This approach is particularly promising for developing sustainable materials that reduce environmental impact while delivering superior energy storage capabilities. This method simplifies the production method and enhances performance, making it a compelling alternative for creating high-efficiency, eco-friendly energy storage solutions. Moreover, this DNA is extracted from biological fish waste which we can turn into an efficient energy source.

## 4. Conclusions

While the concept of using biomass for carbon production is not new, this study aims to simplify the synthesis process and enhance performance by utilizing a unique biological material DNA. By incorporating DNA into the carbon structure, we address some of the challenges associated with traditional carbon sources. Our approach combines a straightforward one-step hydrogel carbonization method with the advantages of nitrogen-doped carbon, providing a practical and cost-effective alternative to more complex and expensive methods such as those used for graphene or carbon nanotubes (CNTs). The goal is to offer fresh insights into the practical applications of these materials and contribute to the development of new kinds of sustainable and efficient energy storage solutions.

## Data Availability

The data that support the findings of this study are available from the corresponding author upon reasonable request.

## Supplementary Materials

The following supporting information can be downloaded at: https://www.mdpi.com/article/10.3390/nano15040304/s1, Figures S1 and S2: Surface morphological and elementary analysis of the synthesized DC-x compounds. Figure S3: BET analysis of the DC-x electrodes. Figure S4: (a) XRD patterns of the synthesized DC-x compounds and (b) XPS analysis of DC-900. Figure S5: Raman spectroscopy of the synthesized DC-x electrodes. Figure S6: Depicted various electrochemical characterization results for the DC-800 and DC-1000 electrodes. Table S1: It presents a summary of previous reports on various resource-derived carbonaceous electrodes. [32,33,34,35,36,37,38,39,40,41,42] are cited in supporting information.
