# Supplementary material for: Porous Carbon Electrode Made of Biomass DNAs for High-Efficiency Quasi-Solid-State Supercapacitor
Source: Nanomaterials (Basel). 2025 Feb 17;15(4):304. doi: 10.3390/nano15040304 (PMC11858159; doi:10.3390/nano15040304)
Supplement: Supplementary file 1 [file nanomaterials-15-00304-s001.zip › nanomaterials-3417701-supplementary.pdf]

Supporting Information

Porous carbon electrode made of biomass DNAs for high-efficiency quasi-solid-state supercapacitor

Samanth Kokkiligadda*, Surya Kiran Ampasala, Yeonju Nam, Jeonghun Kim, Suk Ho Bhang, Soong Ho Um*

Correspondence: samanth213@skku.edu and sh.um@skku.edu, 2066 Seobu-ro, Jangan-gu, Suwon-si, Gyeonggi-do 16419, Korea

Contents

Supporting Figures S1&S2 | Surface morphological and elementary analysis of the synthesized DC-x compounds.

Supporting Figure S3 | BET analysis of the DC-x electrodes.

Supporting Figure S4 | (a) XRD patterns of the synthesized DC-x compounds and (b) XPS analysis of DC-900.

Supporting Figure S5 | Raman spectroscopy of the synthesized DC-x electrodes.

Supporting Figure S6 | Depicted various electrochemical characterization results for the DC-800 and DC-1000 electrodes.

Supporting Table S1 | It presents a summary of previous reports on various resource-derived carbonaceous electrodes

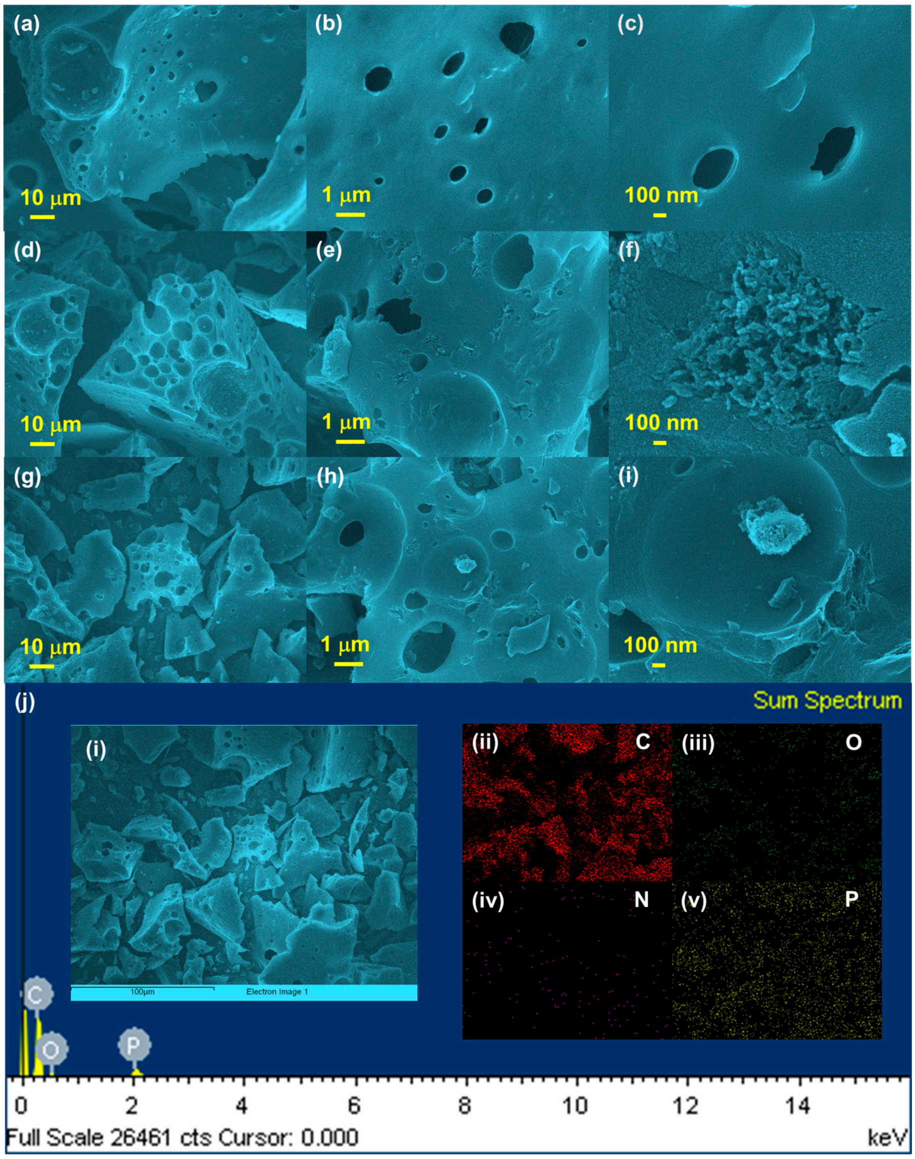

Figure S1. Surface morphological analysis of the synthesized DC-x compounds. FE-SEM images at various magnifications (a-c) DC-800, (d-f) DC-900, and (g-i) DC-1000. (j) EDX spectrum of DC-900. The inset of (j) includes (i) a layered image and (ii-v) elemental mapping images.

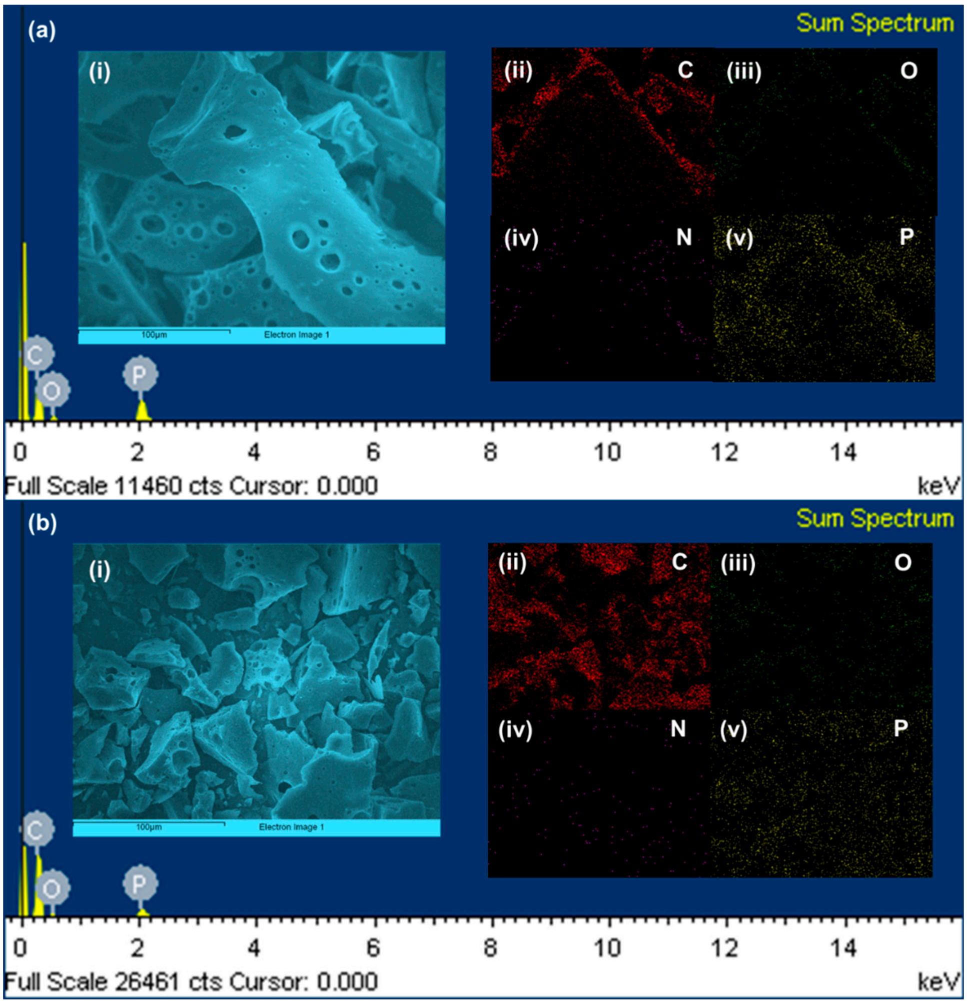

Figure S2. EDX and elemental mapping of DC-800 and DC-1000. (a) EDX spectrum of DC-800. The inset of (a) includes (i) a layered image and (ii-v) elemental mapping images. (b) EDX spectrum of DC-1000. The inset of (a) includes (i) a layered image and (ii-v) elemental mapping images.

Figures S1-2 illustrates the surface morphology and EDX analysis of the DC-900, and DC-1000 electrode materials, respectively. In Figure S1a-c, DC-800 smooth surface with small pores appeared, while an increase in temperature to 900°C yields micro size porous structure for DC-900 (Figure S1d-f). However, at 1000°C, smooth surface disappears giving rise to meso, micro porous structure as depicted in Figure S1g-i. The porous surface morphology of DC-900 contributes to an elevated surface-to-volume ratio, thereby amplifying surface accessibility. To investigate the retention of the naturally occurring sugar-phosphate and hydroxyl groups from DNA and the diffusion of ions, EDX spectroscopy was conducted on DC-900 and all samples. As depicted in Figure S1j, the EDX spectrum across the scanning area confirms the presence of elements such as C, O, N, and in DC-900, and in all samples. Figures S2a(i-v) and Figure S2b(i-v) display the elemental mappings of the detected elements for DC-800 and DC-1000, respectively.

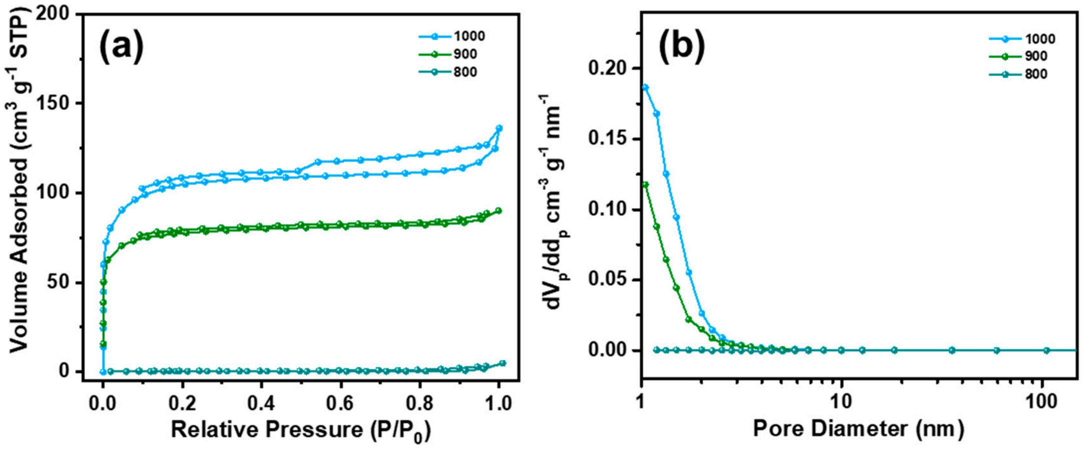

Figure S3. BET analysis of the DC-x electrodes. The analysis of specific surface area and pore size distribution is pivotal for understanding electrode electrochemical properties. The fabricated samples underwent N2 sorption isotherm analysis. Figure a depicts the isotherms of DC-900 and DC-1000 showing a type-III isotherm plot, indicating a primarily mesoporous nature. A slight deviation between the desorption and adsorption curves creates a hysteresis loop, suggesting the presence of low capillary condensate within the pores. The specific surface area of DC-900 exhibits a type-I isotherm with a small hysteresis loop at medium pressure, indicating plentiful micropores and a higher specific surface area of 326.26 m^2 g^{-1}. Figure b illustrates the BJH plots of DC-900 and DC-1000. It shows a sharp peak at ~ 2 nm, suggesting a uniform pore size distribution with extremely small micropores.

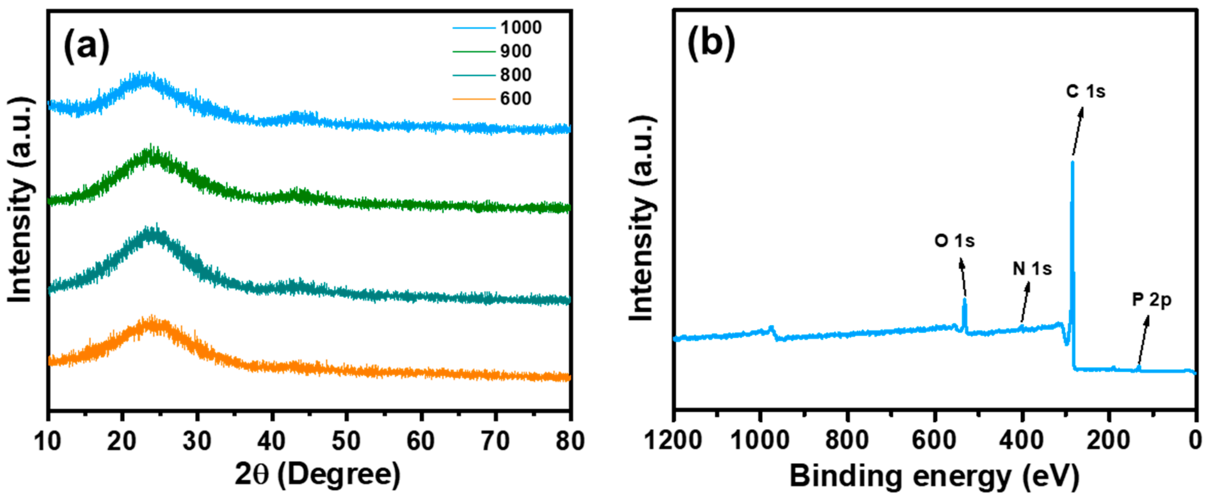

Figure S4. (a) XRD patterns of the synthesized DC-x compounds and (b) XPS analysis of DC-900.

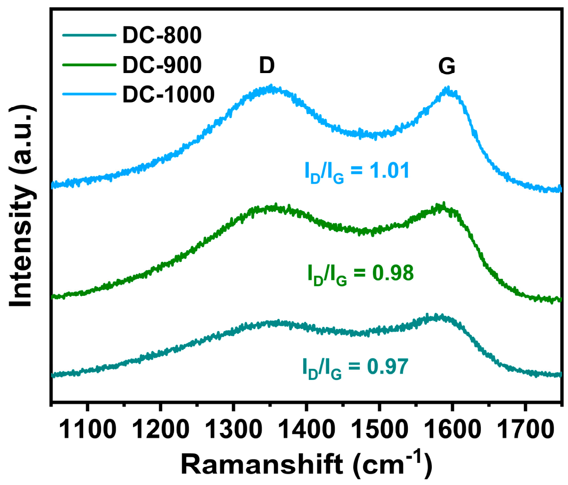

Figure S5. Raman spectroscopy of the synthesized DC-x electrodes.

The DC-x samples were analyzed using Raman spectroscopy, which revealed two distinct peaks around 1355 cm^{-1} and 1590 cm^{-1}, corresponding to the D and G bands, respectively figure S6. The G band is associated with the E2g mode of sp^2 hybridized carbon atoms, while the D band represents the A1g vibration mode, which arises from defects in the carbon structure. The DC-900 electrode with an I_D/I_G ratio of 0.98, demonstrates an ideal balance between graphitization and defect density. This ratio reflects a well-ordered carbon structure, enhancing electrical conductivity, while the remaining defects provide sufficient active sites for pseudo capacitance. Compared to DC-1000 (I_D/I_G = 1.01), the DC-900 electrode exhibits a more developed graphitic structure and lower defect density, resulting in improved transport charge and rate performance. In contrast, the DC-800 electrode (I_D/I_G = 0.97), although highly graphitized, shows slightly reduced pseudo capacitance. DC-900 offers the best combination of energy storage, cycling stability, and charge/discharge efficiency, making it the most effective material for supercapacitor applications.

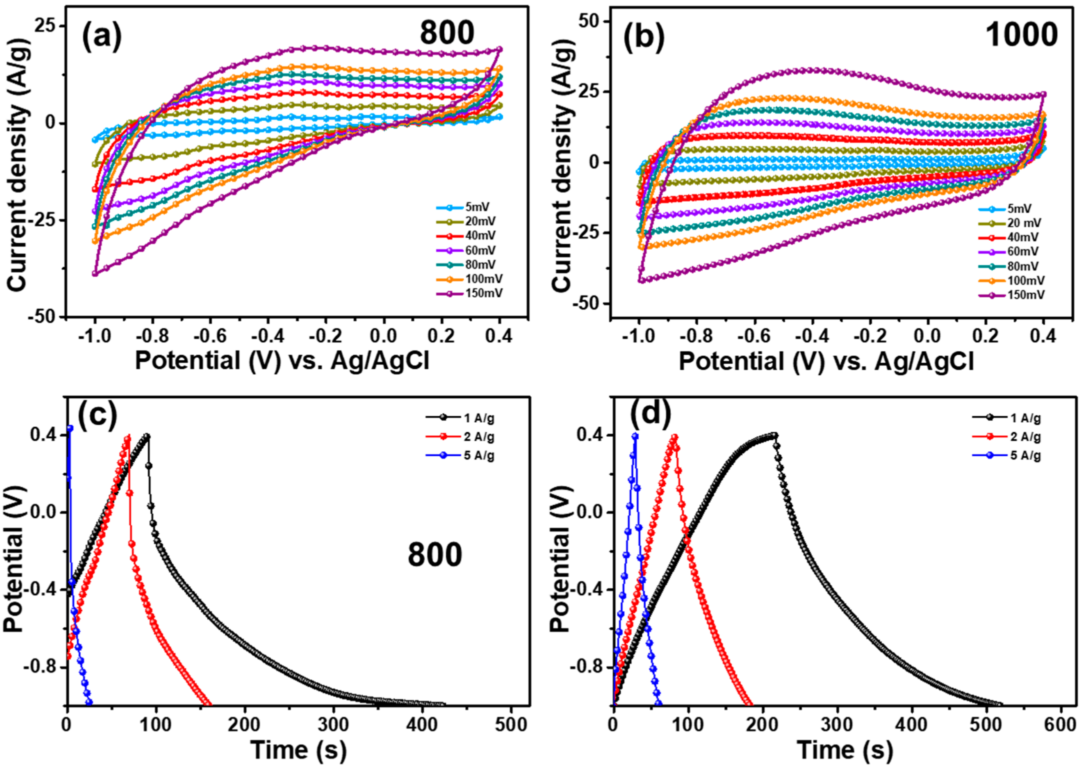

Figure S6. Depicted various electrochemical characterization results for the DC-800 and DC-1000 electrodes: (a,b) Cyclic voltammetry (CV) at different scan rates for the DC-800and DC-1000 electrodes and (c,d) Comparative galvanostatic charge-discharge (GCD) curves at a different current density.

Table S1. It presents a summary of previous reports on various resource-derived carbonaceous electrodes.

**Biomass-derived carbons**	**Electrolyte concentration**	**Specific capacitance ($F\ g^{-1}$)**	**Reference***
Onion	6M KOH	127	32
Natural cellulose	5M KCl	107	33
Pig skin	6M KOH	287.1	34
Humic acids	6M KOH	209	35
Perilla frutescens	6M KOH	270	36
Coconut shell	6M KOH	228	37
Withered rose flower	KOH/KNO_3	350	38
Sodium lignosulfonate	6M KOH	370	39
Garlic skin	6M KOH	427	40
Waste eucalyptus bark	1M H_2SO_4	483	41
Bacterial cellulose	6M KOH	318	42
Pure DNA	1M KOH	563.34	Our Work

Table 1 particularly relates to comparisons under various electrolyte conditions. The difference in electrolyte composition and concentration can indeed have a significant impact on supercapacitor performance. Standardizing electrolyte conditions for all tested materials will allow for more accurate comparisons in practice. However, under the assumption that it strives to achieve the best performance on its own, it is recognized that at present, this comparison serves as an initial reference for identifying potential performance changes between materials, while more controlled studies are needed for accurate performance evaluation.

References

[32] M. D. Mehare, A. D. Deshmukh, S. J. Dhoble, *J. Mater. Sci.* **2020**, 55, 4213–4224.

[33] L. Jiang, G. W. Nelson, S. O. Han, H. Kim, I. N. Sim, J. S. Foord, *Electrochim. Acta* **2016**, 192, 251–258. DOI: https://doi.org/10.1016/j.electacta.2015.12.138

[34] Y. Zhou, J. Ren, Y. Yang, Q. Zheng, J. Liao, F. Xie, W. Jie, D. J. Lin, *Solid State Chem.* **2018**, 268, 149–158. DOI: https://doi.org/10.1016/j.jssc.2018.08.041

[35] Y. Zhu, M. Chen, Y. Zhang, W. Zhao, C. Wang, *Carbon* **2018**, 140, 404–412.

[36] B. Liu, M. Yang, D. Yang, H. Chen, H. Li, *Electrochim. Acta* **2018**, 272, 88–96. DOI: https://doi.org/10.1016/j.electacta.2018.04.001

[37] J. Mi, X. R. Wang, R. J. Fan, W. H. Qu, W. C. Li, *Energy and Fuels* **2012**, 26, 5321–5329. DOI: https://doi.org/10.1021/ef3009234

[38] A. Khan, R. Arumugam Senthil, J. Pan, Y. Sun, X Liu, *Batter. Supercaps* **2020**, 3, 731–737. DOI: https://doi.org/10.1002/batt.202000046

[39] J. Pang, W. Zhang, J. Zhang, G. Cao, M. Han, Y. Yang, *Green Chem.* **2017**, 9, 3916–3926. DOI: https://doi.org/10.1039/C7GC01434A

[40] Q. Zhang, K. Han, S. Li, J. Li, K. Ren, *Nanoscale* **2018**, 10, 2427–2437. DOI: https://doi.org/10.1039/C7NR07158B

[41] K. Li, Z. Liu, X. Ma, Q. Feng, D. Wang, D. Ma, *New J. Chem.* **2023**, 47, 5316–5329. DOI: https://doi.org/10.1039/D2NJ06060D

[42] L. Ma, R. Liu, H. Niu, L. Xing, L. Liu, Y. Huang, *ACS Appl. Mater. Interfaces* **2016**, 8, 33608–33618. DOI: https://doi.org/10.1021/acsami.6b11034
